# Erie County Medical Society

**Published:** 1887-07

**Authors:** 


### Society Reports.

### ERIE COUNTY MEDICAL SOCIETY.

### Semi-Annual Meeting held June 15, 1887, at Y. M. C. A. Building.

The meeting was called to order at 10.30 o'clock by President A. C. Strong, M.D. After the reading of the minutes, the following applicants for membership were duly elected: Drs. Jacob Goldberg, Harry A. Wood, Bina A. Potter, J. S. Meindenbauer, George W. Cutter, William A. Hoddick, George Palmer, Julius Pohlman, and Roland Miller.

In absence of Dr. Barker, Dr. Hopkins presented to the society a copy of the proposed by-laws, prepared by the committee. Upon examination, it was found that a clause relating to the examination of all students desiring to enter members' offices by the Board of Censors as to their education in English, as well as Latin and Greek, had been omitted from the new by-laws. Dr. Hopkins claimed that it was an error of the printer, and advocated its restoration. Some of the members thought it was unjust to require a high degree of literary scholarship as a pre-requisite to admission to the study of medicine, and protested against the clause requiring six months' study of Latin. After much spirited debate, the section under discussion was adopted as one of the by-laws.

Another section providing that five members should constitute a quorum to do business was objected to, and the number was raised to fifteen.

Dr. Storck then presented the following report from the Board of Censors:

By some articles that appeared in the local press, and also the statements made to us by members of this society and the Civil Service Examiners, the attention of your board has been called to the examination of applicants for he position of City District Physician of the fifth district made by the Civil Service Commission, and also the results and facts thereby developed; that we deemed it our duty to take official action thereon, and report the same to this society.

Through personal inquiries we have gained reliable information that all the applicants were graduates of recognized medical schools and legally registered at the County Clerk's office, but nearly all have so signally failed in the attempt to pass the examination, and have shown such gross ignorance of the medical science and a total want of a literary or classical education, their written work having been so poor and defective in spelling that at the first examination not one of them reached the lowest standard of sixty, set down by the commissioners, and all were rejected. The Board of Health in its mercy passed a resolution requesting the examiners not to publish the names of those physicians, as it might do them injury in their private practice. These facts have been published by the local press.

As a sample of the "good English academical education" as required by law, but which rather shows the blissful ignorance of the writer of the English language — say nothing of Latin and Greek—we annex an extract of a letter written by one of those applicants, a graduate of one of our medical schools, which was sent to the board. Leaving off names, the copy is verbatim.

"Mrs. S. B. ——— : I want you to git som one to go on that Bond for you at once as it is necery for me to with draw myne at once, if you due not git any one there will be some trubell as I have got to with draw and I want you to Setell your Bill with me at once or I will have to sue you for the amount and git judment.

I can not wait on you any longer than tomorrow at 10 o'clock A. M.

Yours, ets., DR. B."

The author of this literary production is no foreigner; he is a native of the State of Ohio; he had the opportunity this country offers to acquire a common school education, which he certainly should have had before he became a student of medicine and acquired the title of M. D.

Some of the written examinations before the Civil Service Commissioners were in about the same style, if not worse.

Taking these deplorable facts into consideration, your board has come to the conclusion that the by-laws of the Medical Society of Erie County have been disregarded and flagrantly violated by some of its members in having admitted to their offices as students of medicine such applicants, without the necessary certificates of the Board of Censors, as prescribed for in our by-laws; or, on the other hand, the State law, the charter of the medical college has been violated through its faculty granting a diploma to a candidate who has not produced a certificate of his preceptor, setting forth that he has studied medicine with him, in compliance with the requirements embodied in the charter of every medical college in this State.

A city ordinance relating to the appointments for the offices of district physicians by the Board of Health provides that "the appointee must be a member in good standing of the medical profession."

Your board holds that, while a person holding a diploma of a recognized medical school and having made the required registry, is recognized by the law of 1880 as a legal physician, he is not, in pursuance of a former law, which is as claimed by able lawyers of New York, still in force, a legal practitioner, if he has not become a member of the county medical society where he resides, as provided by a section of said medical law. He could, therefore, not be a member in good standing of the medical profession and would not be eligible for those offices.

A bill that has emanated from this society has been pending in the State Legislature the last two sessions: "To Create a State Board of Medical Examiners."

The existence of such a commission, empowered with the authority as the act provides, would probably stop the abuses of medical colleges graduating annually a large number of candidates that are incompetent and unqualified to enter upon the practice of medicine. It would elevate the standard of the medical profession in this State; but by some persons, actuated by mercenary motives and self-interests, the passage of said bill has until now been prevented. Our legislators seem to hold these personal interests higher than the interests of the people and the medical profession. The Board of Censors would, therefore, propose to take the next step in the way of reform, hoping that other county medical socteties in this State will follow the example.

The following resolutions were offered for adoption by the society:

"*Resolved*, That the committee on membership is hereby directed to strictly adhere to the provisions of Sec. 6, Art. VI. of our by-laws."

If, after careful examination of the credentials of the applicant, a majority of the committee come to the conclusion that his diploma was acquired by him, or granted to him in violation of the State Medical Law, and the by-laws of this society, then the application shall be rejected by the committee and reported to the society. Also:

"*Resolved*, That the Board of Censors is hereby directed to report at the next annual meeting and at every subsequent regular meeting of the society, the name of any member known to the Censors who shall, during the time, have admitted to his office as a student of medicine any applicant without having received of him the necessary certificate of the Board of Censors, as provided for by Sec. 5, Art. VI. of our by-laws."

The board would also recommend the following resolution for adoption:

"*Resolved*, That it is the sense of this society that the present mode of examination of applicants for the offices of District City Physicians and Health Inspectors by the Civil Service Commission and the appointment by the Board of Health on their recommendation is the only proper way to take these offices out of politics, to exclude ignorant and incompetent applicants and to secure the services of qualified physicians to the city poor. In expressing our approval of the action of the Board of Health, we would recommend to the same that the appointment of Health Physicians of Buffalo be also placed under the same rule of the Civil Service law."

Dr. John Cronyn objected to the expression, "The Board of Health in its mercy suppressed the names." As a member of the Civil Service Commission, he would state that the names of those who undertake the examination and fail to pass are never officially known. Candidates and their papers are numbered.

The report was then adopted.

Doctor Storck had lost his manuscript, and so made an additional verbal report as follows: He said that several times during the past six months complaint has been made to the board of the violation of the rule of the society against traffic in nostrums by one of its members.

Dr. Storck said that the offending member is sending out four-page circulars, the title page of which reads as follows:

" A Cure for Diphtheria, Membraneous Croup, Scarlet Fever, and all forms of Sore Throat. Prepared by John L. C. Cronyn, M. D."

Dr. J. L. C. Cronyn was notified by the Board of Censors of the complaint against him, and told that he would be given a hearing by the society if he had anything to say in extenuation of his unprofessional conduct, but failed to appear.

Dr. John Cronyn arose and said that he desired it distinctly understood that he was not the John Cronyn referred to in the report, and that he had during his long professional life in the city never sustained such a course. He had nothing whatever to do with the present case. (Loud and prolonged applause.)

Dr. Green moved that the matter under discussion be laid on the table for six months, in the hope that in the interim it would adjust itself.

Dr. Hopkins said that if the society had a disagreeable duty to perform, the sooner it was done the better. He believed that some declaration of intention from some one qualified to speak for Dr. John L. C. Cronyn should be made, or else the society's censure should rest upon the offender and the fine be imposed. Dr. Fell moved that Dr. John L. C. Cronyn be censured and fined.

The Board of Censors have also come into possession of a pamphlet whose title page bears the words: "A Guide for the Road to the Goal of Health, with compliments of J. S. Langdon, M. D., Buffalo." The subject matter of the pamphlet consists of descriptions of private diseases and a declaration of Dr. Langdon's power to heal them.

The Board of Censors made inquiries at the Buffalo Post-office and were informed that the mail for Dr. J. S. Langdon was placed in a lock-box rented by Dr. J. S. Armstrong.

Dr. Armstrong was forthwith summoned before the Board and asked to give an explanation of his unprofessional conduct. He admitted mailing 3,500 of the pamphlets to persons residing outside the city, and claimed that the business was one which he would continue.

Dr. C. C. Frederick asked permission to state some instances of unprofessional conduct on the part of Dr. John L. C. Cronyn in addition to the charge preferred by the Board of Censors. He related three instances in which, while he was attending children for throat diseases, Dr. J. L. C. Cronyn paid unsolicited visits to the same patients and left bottles of his syrup.

The motion to censure Drs. J. L. C. Cronyn and Armstrong was then carried without dissent.

The society then adjourned.

